# Moving from a pilot study to large pragmatic trial in primary care settings: A study on acute rhinosinusitis

**DOI:** 10.1017/cts.2025.10175

**Published:** 2025-11-06

**Authors:** Daniel Merenstein, Bruce Barrett, Sebastian T. Tong, Aleksandra E. Zgierska, David P. Rabago, Derjung M. Tarn, Keisha Herbin Smith, Gabriela Villalobos, Danielle Schramm, Cameron Casey, Tina P. Tan, Charles R. Fencil, Stephen Fernandez, Mihriye Mete, Nawar Shara, Nicholas Franko, Jessy Sparenborg, Alex H. Krist

**Affiliations:** 1 Georgetown University Medical Center, Department of Family Medicine, Washington, DC, USA; 2 University of Wisconsin - Madison, Department of Family Medicine and Community Health, Madison, WI, USA; 3 University of Washington, Department of Family Medicine, Seattle, WA, USA; 4 Penn State College of Medicine, Department of Family and Community Medicine, Hershey, PA, USA; 5 David Geffen School of Medicine at UCLA, Department of Family Medicine, Los Angeles, CA, USA; 6 Virginia Commonwealth University, Department of Family Medicine and Population Health, Richmond, VA, USA; 7 MedStar Health Research Institute, Columbia, MD, USA

**Keywords:** Point of care test, acute rhinosinusitis, clinical research, antibiotic stewardship, primary care

## Abstract

**Background:**

Acute rhinosinusitis is one of the most common conditions seen in primary care. One in seven adults are diagnosed with ARS annually, resulting in one in five of all antibiotic prescriptions. Yet there has been limited research comparing the effectiveness of widely used treatments such as antibiotics and nasal steroids. Conducting such a trial in the context of decades of established practice poses unique challenges.

**Methods:**

A feasibility phase was conducted with continuing feedback to provide refinement and guidance regarding the design of a large-scale, pragmatic randomized controlled trial. The pilot trial assessed the ability to enroll, retain, and evaluate adherence to the intervention and assessment protocols.

**Results:**

The feasibility phase allowed us to seek input from patients and experts. This resulted in changes pre and post pilot that will impact the full study. *A priori* enrollment targets for the pilot were achieved, and with high adherence rates. In total, 373 patients were pre-screened and 140 patients were enrolled participants. Adherence to data collection via the daily diary was 93% throughout the study, with 95% completing their diary on the day of the primary outcome, 3 post-randomization.

**Conclusion:**

Expert panels and a patient advisory committee recommended critical changes to our study design. Stakeholder engagement is a key component of this funding source and was widely used throughout the 18-months. An achieved primary goal of the feasibility phase was to evaluate recruitment and study methods prior to implementing a large clinical trial that requires significant resources.

## Introduction

The Patient-Centered Outcomes Research Institute (PCORI) has funded pragmatic clinical trials since 2014 [1]. Consistent with the mission of PCORI to help patients and clinicians make informed healthcare decisions and improve healthcare delivery and outcomes, these trials represent comparative clinical effectiveness research (CER). The trials are designed to be large, real-world pragmatic studies, that answer questions often not addressed by other funders but nevertheless critical to patients and patient care. Before funding these large, expensive trials, PCORI often requires an 18-month feasibility phase, including a pilot study, the successful completion of which is a requirement prior to the PCORI’s approval to proceed to the full study. We are reporting on how the feasibility phase advanced our understanding and informed the design of our “Nasal Irrigation, Oral Antibiotics, and Subgroup Targeting for Effective Management of Acute Sinusitis” (NOSES) trial.

The NOSES study aims to effectively treat acute sinusitis while reducing inappropriate antibiotic use and growing bacterial resistance. Global overuse of antibiotics increases antibiotic resistance and unnecessary medication-related adverse events [2]. The COVID-19 pandemic has underscored the importance of proper diagnosis and treatment of respiratory infections in primary care. Unfortunately, acute rhinosinusitis (ARS) is one of the most common conditions that is inappropriately treated with antibiotics, although ARS is usually caused by viruses [3]. In the U.S., one in seven adults is diagnosed with ARS every year, across 30 million office visits, resulting in one in five of all antibiotic prescriptions and accounting for over $11 billion in direct annual costs [4–10]. Because ARS is so prevalent and the impact of inappropriate antibiotic use is so widespread, the populations and health decisions affected by this research will likely have a major impact on public health.

Reducing inappropriate prescribing for ARS is crucial to limiting the development and spread of antibiotic resistance [11]. The World Health Organization has identified the overuse of antibiotics and subsequent resistance as a top public health concern, while the United Nations convened a high-level meeting to coordinate approaches to address the root causes of antimicrobial resistance [12], the fourth health issue to ever be addressed by the General Assembly [13]. Inappropriate use of antibiotics is not just a societal issue but an individual issue as well. Our research group and other researchers have shown significant microbiome and metabolic changes that may have long-term health impacts on individuals [14–18]. Per the Centers for Disease Control and Prevention (CDC), antibiotics are one of the most common reasons for emergency department visits for adverse drug events, responsible for 16% of all adverse drug reactions [19].

ARS is often inappropriately treated because of the difficulty of distinguishing viral from bacterial ARS and because of the lack of point of care tests or evidence-based clinical prediction rules. Other infections in primary care, such as pharyngitis, COVID-19, urinary tract, or pneumonia, have clinical and/or laboratory tools readily available to distinguish viral from bacterial infections to guide antibiotic prescribing [4,20–23]. A large study is needed to provide this type of data to guide ARS’ treatment with antibiotics, the type of study supported by PCORI’s comparative effectiveness pragmatic trial initiatives. Accordingly, little funding has been expended on ARS, with the most recent Cochrane review finding only 15 studies on ARS care, in comparison to the last Cochrane review on Vitamin D and mortality that included 159 relevant articles [4]. We view this as part of the larger problem of too little primary care research, with only 0.3 percent of National Institutes of Health (NIH) funding supporting primary care-driven research [4,24].

To better understand ARS, a common disease, and provide clinicians with the tools to determine the comparative benefits of antibiotics versus the most commonly used non-antibiotic treatment, intranasal corticosteroids (INCS), we designed NOSES, a parallel 4-arm randomized controlled trial (RCT). All enrolled patients were given options for supportive care, and supplies for nasal saline irrigation. NOSES will evaluate disease-specific quality-of-life outcomes that are patient-reported and -centered and approved by the study’s patient and other stakeholder partners who have advised on the project design and implementation. The specific aims of the NOSES trial are (1) To compare, through patient-reported outcomes, the efficacy of oral antibiotics (amoxicillin-clavulanate), and INCS, for clinical improvement of ARS symptoms among adults who did not improve with supportive care alone during the first 10 days of their symptoms. We expect approximately 60% of individuals will not improve with supportive care. (2) To identify which subgroups of participants benefit most from oral antibiotics (amoxicillin-clavulanate) versus INCS. Potential subgroups of interest, suggestive of bacterial infection, include participants with (a) an elevated C-reactive protein (CRP) level, (b) double-sickening, defined as, “Have you had worsening of sinus symptoms after initial improvement,” (c) evidence of nasal discharge purulence on clinical examination, (d) change in smell (cacosmia), (e) pain in the teeth, or (f) provider’s clinical impression [21,25–30]. These subgroups are suggestive of bacterial infection [28]. (3) To identify which participant subgroups improve with supportive care and do not require antibiotics or INCS. Clinicians could recommend any supportive care but all patients were provided supplies and instructions for saline nasal irrigation.

We report here on the results of our pilot study, which was not powered to find between-group differences, but designed to help us demonstrate feasibility and better understand what is needed to successfully complete the full-scale study projected to start in January 2025. The purpose of this manuscript is to highlight the value of a feasibility phase and discuss how it can influence a large pragmatic study. In order to truly conduct a pragmatic trial, it is helpful to get input from a variety of sources. The primary goal of the pilot study was to assess the ability to enroll (target *n* = 144), randomize and retain (*n* ≥ 68), and evaluate adherence to the interventions (greater than 71%) and assessment protocols (greater than 80%). Another important goal of the pilot was to obtain and incorporate feedback from patients, experts, and other stakeholders.

## Methods

### Study overview and regulatory approval

The feasibility phase centered on two major activities: (1) engagement with patient, clinician, and scientific stakeholders to garner qualitative input to help operationalize study plans; and (2) a pilot RCT to assess the capacity to enroll, retain, and adhere to the protocol.

A single IRB was used, BRANY #23-02-622, and the study was registered prior to recruitment at ClinicalTrials.gov, #NCT06076304. This study followed the Consolidated Standards of Reporting Trials (CONSORT) reporting guideline for clinical trials, and the full trial protocol is currently under review.

### Design

#### Focus groups

In the first few months of the feasibility phase, 3 focus group sessions were held; one session with primary care physicians, one with other primary care clinicians (physician assistants and nurse practitioners), and one with clinic personnel (nurses, medical assistants, front desk, laboratory, etc.). Two semi-structured interview guides with open-ended questions were developed: one tailored for clinicians which focused on the medical aspects of the trial design; one for nonclinical personnel that focused on patient recruitment and staff engagement. Additionally throughout the pilot, we had longitudinal oversight and feedback from our expert and patient advisory panels resulting in advice from these panels on how to adapt the study design both before and after the pilot was conducted.

#### Engagement activities

**Figure 1.**
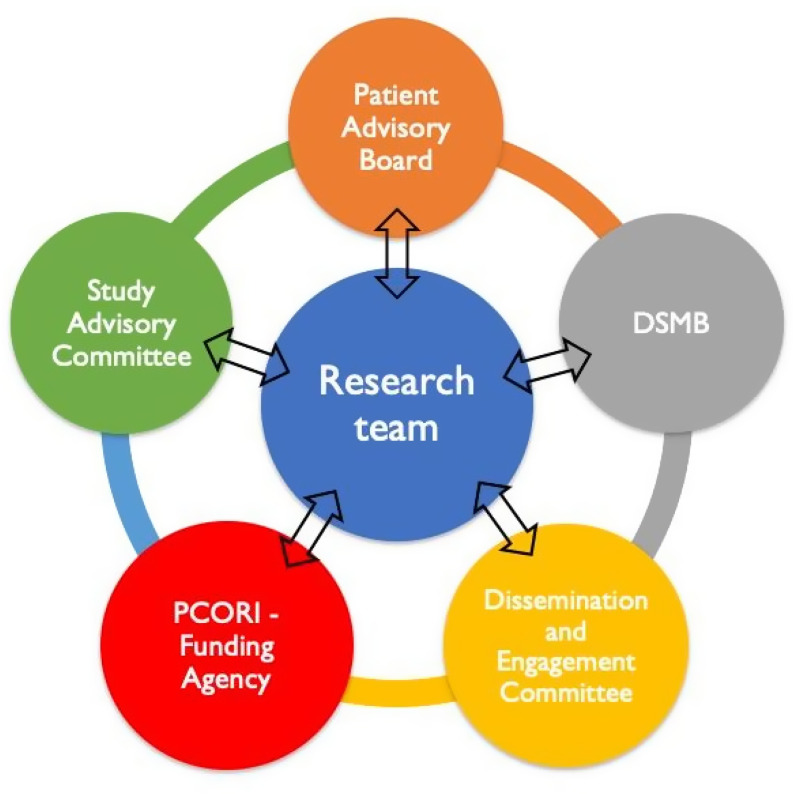
Committees.

Early in the 18-month feasibility phase we set up 4 committees; a Patient Advisory Board (PAB), a Data Safety and Monitoring Board (DSMB), a Study Advisory Committee (SAC), and a Dissemination and Engagement Committee (DEC). The SAC and DEC includes experts and stakeholders interested in primary care and antibiotic stewardship. They have a variety of expertise in different medical fields, statistics, and clinical trial implementation. The PAB includes 2 representatives from each of the 6 clinical sites and has been instrumental in ensuring our questions, outcomes and approaches are amenable to patients. All of these groups work together and information from each is shared with the others, please see Figure 1. We recruited over 30 committee members representing a diverse range of stakeholder groups. These committees met a combined 14 times throughout the study.

#### Pilot RCT setting and participants

**Figure 2.**
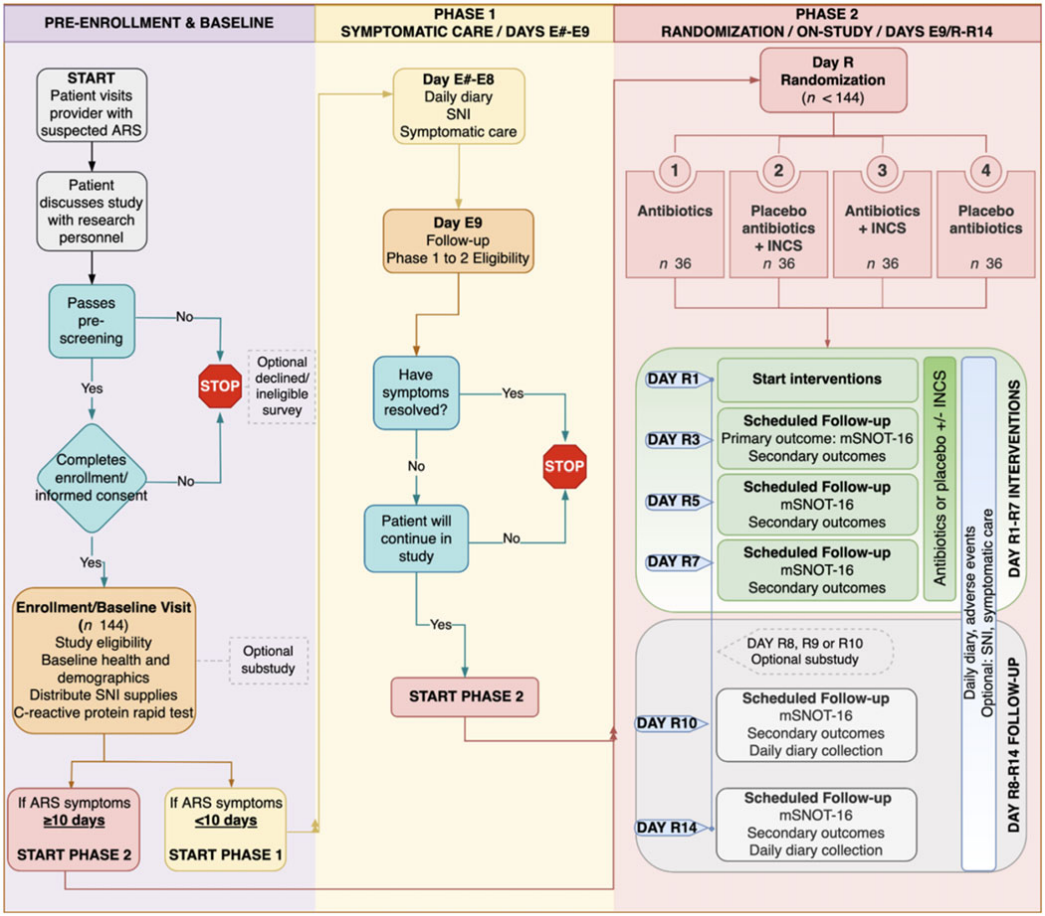
Participant flow diagram for pilot study.

A scaled down (unpowered) version of the proposed full-scale protocol was designed to demonstrate whether the target accrual rates for enrollment, retention, and adherence were achievable at the selected clinic sites. Other aspects of the patient experience and impact on clinic workflow were assessed. A schematic of the pilot (Figure 2) shows a timeline of the initial clinician visit, recruitment, enrollment, randomization/allocation, intervention, and follow-up periods, starting from the supportive care period through the final day of the study (day 14). Participants were enrolled into the study based on how many days they had ARS symptoms; if symptoms were present for fewer than 10 days prior, participants started in the supportive care phase – days are referred to as E on the diagram – until E9. All participants were generally healthy, English speakers between 18–65 years of age. Supplemental Table 1 includes the full inclusion/exclusion criteria. We recruited patients from 6 diverse practice-based research networks based at Georgetown University, Virginia Commonwealth University, Penn State College of Medicine, the University of Wisconsin – Madison, the University of Washington, and the University of California, Los Angeles.

#### Interventions and blinding

Participants who were sick for 9 days or less at enrollment were recommended supportive care. If they did not improve by day E9, or had >9 days of symptoms at enrollment, they were randomized to one of four groups: group one received antibiotics, group two received placebo antibiotics plus INCS, group three received antibiotics plus INCS, and group four received placebo antibiotics. All participants received a Neti pot, salt packets, and instructions for SNI. Participants were followed for 14 days after randomization.

The study antibiotic and dose was amoxicillin-clavulanate 875 –125 mg, taken twice daily for 7 days, as recommended by the aforementioned IDSA guideline. The INCS spray was 32 mcg of budesonide in each metered spray administered in 2 sprays per nostril, once daily. Both were provided to each site by Doyle’s Pharmacy of Houston, TX. The pharmacy over-encapsulated the amoxicillin-clavulanate pills and prepared the matching placebo. We utilized a masking procedure of different unique numbers to ensure proper randomization, blinding, allocation, and implementation of these research procedures. We used permuted block randomization by site with variable block sizes (4, 8, or 12) to enroll patients.

#### Primary outcome measurements

As previously mentioned, our primary goal were to obtain expert and patient input of the process and experience and to adapt the trial to inform study design. The objectives of the pilot trial were to evaluate if target rates of enrollment (target *n* = 144), randomization and retention (n≥68) were achievable.

The primary outcome of the full-scale study, patient improvement, is a disease-specific quality-of-life score (the modified Sinonasal Outcome Test (mSNOT-16), which was measured at Day 3 after randomization. Day 3 was chosen based on the Infectious Diseases Society of America guidelines that recommend changing treatment if no improvement after 3 days [31]. The mSNOT-16 is validated but has not been used often; another aim of the feasibility phase was to determine if the scale is an appropriate primary outcome measure for the full-scale study.

#### Adherence assessment

We assessed study drug adherence through participant self-reports via the daily diary and follow-up interviews. As part of the daily diary, participants recorded the number of capsules taken per day, and use of INCS. Participants were informed to save all unfinished drugs and to report how many capsules were left at the end intervention period. On day 8, participants were asked to photograph the bottle showing the remaining capsules and upload it to the study database. We assessed compliance via a continuous variable; the participant was deemed compliant if 10 or more capsules (out of 14 maximum) of amoxicillin-clavulanate were taken. Participants were also compliant if they used they study INCS for at least 5 of the 7 maximum days. Participant retention throughout their illness and diary completion were also assessed, both with target rates of 80% or higher.

#### Sample size

For the full-scale study to be sufficiently powered, the sample size was determined to be 3,720 participants (enrolled). Based on focus groups and clinician reports, we estimated that 60% of participants would transition to the randomization phase, and 40% would improve or withdraw during the supportive care phase. The sample size for the pilot was based on the 3,720 participants needed for the full study over the total number of recruitment months. The comparable rate was calculated to be 24 participants per site in two months. Thus, the targeted sample size for the pilot was 24 participants for each of the 6 sites, for a total of 144.

### Statistical analysis

Data collected during the pilot were summarized using descriptive statistics, such as means and standard deviations for continuous variables and frequencies and percentages for categorical variables. Bivariate statistics (two-sample t-tests, proportions test, Fisher’s exact test, Chi-square test or Analysis of Variance (ANOVA) were used as appropriate to conduct two-group or four-group comparisons. Linear mixed models were used to analyze longitudinal data for the primary outcome measure mSNOT-16, including time indicator, group indicator, and their interactions with random effects at the patient level to explore trends over time. The mSNOT-16 scores were also compared to the 6-point Patient Global Impression of Change Scale. Stata 17 was used to conduct statistical analyses.

## Results

### Qualitative feedback

Each of the focus groups provided valuable input on different aspects of the trial [32]. A few specific recommendations necessitated further discussion with PCORI, and prompted protocol changes prior to the pilot trial. One such modification was to the earlier exclusion of patients diagnosed with COVID-19 from the trial. The physician focus group properly pointed out that nearly all ARS starts with a virus, and that if patients were still sick with ARS symptoms by day 10, most primary care clinicians would prescribe antibiotics. The protocol was amended to include qualifying patients with positive COVID-19 diagnoses.

In addition to the focus groups, the stakeholder committees (PAB, SAC and DEC) raised suggestions that did not require methodological re-design but that would optimize recruitment and study operations. One significant change was to the follow-up timeline – originally every 3–4 days to daily – and to add an option to complete the diary on a mobile device, as it is now preferred over voice calling. When it became clear that patients and clinicians refer to ARS in many different ways, we updated how we train our research assistants to discuss the study and to be more inclusive in our descriptions.

The PAB was instrumental to the design and language on patient-facing materials, including recruitment flyers, website, and form collection methods. The PAB was highly engaged with the researchers both pre- and post-pilot.

Additional changes were prompted after the study clinicians and committees reviewed the pilot trial data. After reviewing SNI compliance and extensive feedback from the PAB, we were able to refine how saline nasal irrigation will be broached in the next phase. Both the SAC and DEC were strong proponents of adding double-sickening and increasing the age limit to 75 as inclusion criteria to the full-scale study. PCORI agreed with the new inclusion criteria and protocol modifications.

### Recruitment success strategies

Consistent with a pragmatic trial, each site adapted their operational and recruitment plans to best work for their team, partner clinics, and patient population. Some sites had research coordinators physically present at the clinic, and clinic staff alerted them of patients who might be eligible. Others used electronic medical records to identify patients coming into the clinic who may have qualified for inclusion. It also was apparent that some clinics had champions who strongly believed in the study aims and talked to their patients about reasons to be enrolled in the study.

### Pilot study sample

**Figure 3.**
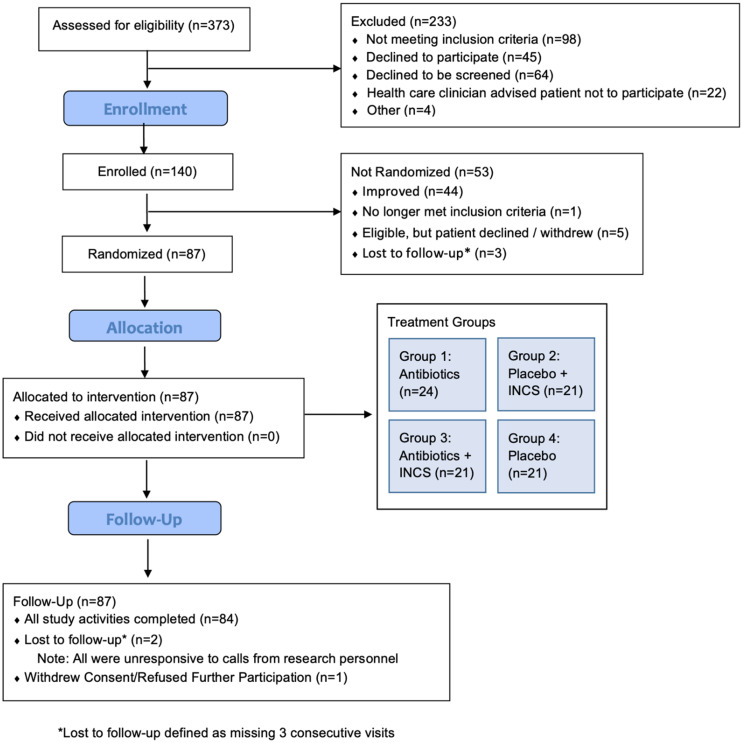
CONSORT flow diagram.

A total of 373 patients were approached or pre-screened at six study sites for the pilot trial; 140 participants were enrolled (see Figure 3). Five sites enrolled the *a priori* sample of 24 participants per site; one site started recruitment later than the other sites and enrolled 20 participants. The sample of 140 participants constituted 97% of our goal of 144 enrolled individuals. Demographic data were collected, as permitted by the IRB, from participants who declined or failed inclusion.

Among the 233 individuals screened but not enrolled, 138 provided their age (59%), 131 reported race (56%), and 144 reported sex (61%). Table 1 shows the distribution of age, sex, and race variables for the 140 enrolled and 233 not-enrolled participants; based on the available data, no statistically significant differences were noted in these characteristics between these two groups. The 140 enrolled participants were, on average, 36.9 years old, with 73% identifying as White and 19% as Hispanic/Latino.

**Table 1.** Demographic characteristics of the participants who enrolled vs. Those who screened out or declined to participate (Not enrolled)

Variable	Total screened *N* = 373	Enrolled *N* = 140	Not enrolled *N* = 233	p-val
**Age (yrs)**	37.7 (12.4)*N* = 276	36.9 (13.3)*N* = 138	38.6 (15.3)*N* = 138	0.33
**Race**				
White	199 (53.4)	94 (67.1)	105 (45.1)	0.94
Black	28 (7.5)	14 (10.0)	14 (6.0)	
Asian	24 (6.4)	12 (8.6)	12 (5.2)	
Other/Unk/missing	122 (32.7)	20 (14.3)	102 (43.8)	
**Sex**				
Female	193 (51.7)	96 (68.6)	97 (41.6)	0.76
Male	90 (24.1)	43 (30.7)	47 (20.2)	

Statistically significant differences were not observed between those who improved on their own before day E9 (*N* = 44), and those who did not improve (*N* = 96) and were subsequently randomized (Table 2). The characteristics of those randomized (total and by group status) are presented in Table 3.

**Table 2.** Overall and by enrollment phase outcome: improved vs not improved (*N* = 140)

	Overall *N* = 140	Improved during phase 1 *N* = 44	Not improved during phase 1 *N* = 96	p-val
Age, mean (SD)	36.9 (13.3) *N* = 138	36.0 (13.0) *N* = 43	37.3 (13.5) *N* = 95	0.59
**Race, N (%)**	*N* = 139	*N* = 43	*N* = 96	0.14
AA/Black	15 (11)	3 (7)	12 (13)	
Asian/South Asian	15 (11)	9 (21)	6 (6)	
Multi-race	3 (2)	1 (2)	2 (2)	
White	101 (73)	30 (68)	71 (75)	
Unknown/missing	5 (4)	1 (2)	4 (4)	
**Ethnicity, N (%)**	*N* = 139	*N* = 43	*N* = 96	0.36
Hispanic/Latino	26 (19)	6 (14)	20 (21)	
**Sex, N (%)**	*N* = 139	*N* = 43	*N* = 96	0.08
Female	96 (69)	26 (59)	70 (74)	
**Education, N (%)**	*N* = 139	*N* = 44	*N* = 95	0.52
High school or less	21 (15)	5 (11)	16 (17)	
Some college/trade	41 (29)	13 (30)	28 (29)	
College degree	40 (29)	11 (25)	29 (30)	
Graduate degree	37 (26)	15 (34)	22 (23)	
**Marital status, N (%)**	*N* = 139	*N* = 44	*N* = 95	0.07
Married	62 (44.3)	14 (32)	48 (50)	
Living with a partner	9 (6.4)	5 (11)	4 (4)	
Single	59 (42)	22 (50)	37 (39)	
Separated	3 (2)	1(2.3)	2 (2)	
Divorced	2 (1.4)	0 (0)	2 (2)	
Widowed	2 (1.4)	0 (0)	2 (2)	
Other/Not reported	2 (1.4)	2 (5)	0 (0)	
**Health insurance, N (%)**	*N* = 136	*N* = 42	*N* = 94	0.09
Yes	132 (94)	39 (89)	93 (97)	
**Income, N (%)**	*N* = 139			
<$50K	29 (21)	14 (32)	15 (16)	0.04
$50=$100K	24 (17)	3 (7)	21 (22)	
>$100K	45 (32)	16 (36)	29 (30)	
Prefer not to answer	41 (29)	11 (25)	30 (31)	
**Employment status, N (%)**	*N* = 139	*N* = 44	*N* = 95	0.18
Employed	106 (76)	31 (71)	75 (78)	
Student/retired	23 (16)	11 (25)	12 (13)	
Unemployed/disabled	10 (7)	2 (5)	8 (8)	

**Table 3.** Overall and by randomization groups (*N* = 87)

	Overall *N* = 87	Antibiotic w/INCS *N* = 21	Antibiotic w/o INCS *N* = 24	Placebow/INCS *N* = 21	Placebow/o INCS *N* = 21
Age, mean (SD)	37.0 (13.4)	32.6 (12.0)	42.5 (14.0)	35.3 (12.9)	36.8 (13.4)
**Race, N (%)**					
AA/black	9 (10)	0 (0)	2 (8)	5 (24)	2 (10)
Asian/South Asian	6 (7)	3 (14)	1 (4)	1 (5)	1 (5)
Multi-race	2 (2)	2 (10)	0 (0)	0 (0)	0 (0)
White	66 (76)	16 (76)	18 (75)	14 (67)	18 (86)
Unknown/missing	4 (5)	0 (0)	3 (13)	1 (5)	0 (0)
**Ethnicity, N (%)**					
Hispanic/Latino	19 (22)	4 (19)	6 (25)	5 (24)	4 (19)
**Sex, N (%)**					
Female	64 (74)	15 (71)	19 (79)	16 (76)	14 (67)
**Education, N (%)**					
High school or less	14 (16)	3 (14)	2 (8)	6 (29)	3 (14)
Some college/trade	25 (29)	7 (33)	8 (33)	5 (24)	5 (24)
College degree	28 (32)	8 (38)	9 (38)	8 (38)	3 (14)
Graduate degree	20 (23)	3 (14)	5 (21)	2 (10)	10 (48)
**Marital status, N (%)**					
Married	45 (52)	9 (43)	13 (54)	12 (57)	11 (52)
Living with a partner	3 (4)	2 (10)	0 (0)	0 (0)	1 (5)
Single	35 (40)	10 (48)	8 (33)	9 (43)	8 (38).
Separated	0 (0)	0 (0)	0 (0)	0 (0)	0 (0)
	2 (2)	0 (0)	2 (8)	0 (0)	0 (0)
Widowed	2 (2)	0 (0)	1 (4)	0 (0)	1 (5)
Other/Not reported	0 (0)	0 (0)	0 (0)	0 (0)	0 (0)
**Health insurance, N (%)**	*N* = 86		*N* = 23		
Yes	85 (98)	21 (100)	23 (100)	20/21 (95)	21 (100)
**Income, N (%)**					
<$50K	14 (16)	5 (24)	2 (8)	2 (10)	5 (24)
$50-$100K	19 (22)	4 (19)	4 (17)	5 (24)	6 (29)
>$100K	28 (32)	8 (38)	9 (38)	7 (33)	4 (19)
Prefer not to answer	26 (30)	4 (19)	9 (38)	7 (33)	6 (29)
**Employment status, N (%)**					
Employed	67 (77)	16 (76)	18 (75)	16 (76)	17 (81)
Student/retired	12 (14)	4 (19)	2 (8)	3 (14)	3 (14)
Unemployed/disabled	8 (9)	1 (5)	4 (17)	2 (10)	1 (5)

Adherence to the assessment protocol was higher than anticipated in our sample size calculations. The overall rate of daily diary completion was 93% throughout the study, and over 95% diary completion on day 3 post-randomization, representing the primary outcome measure endpoint. Daily diary data collection adherence varied between 91% and 100% during the initial phase (between enrollment and randomization) and 90% and 98% post-randomization.

Adherence to the intervention protocol was more mixed. During the study, 39% of participants reported trying nasal saline irrigation at least once. Post-randomization, participants reported over 78% adherence to the antibiotic or placebo capsules and 81% compliance with using INCS.

**Figure 4.**
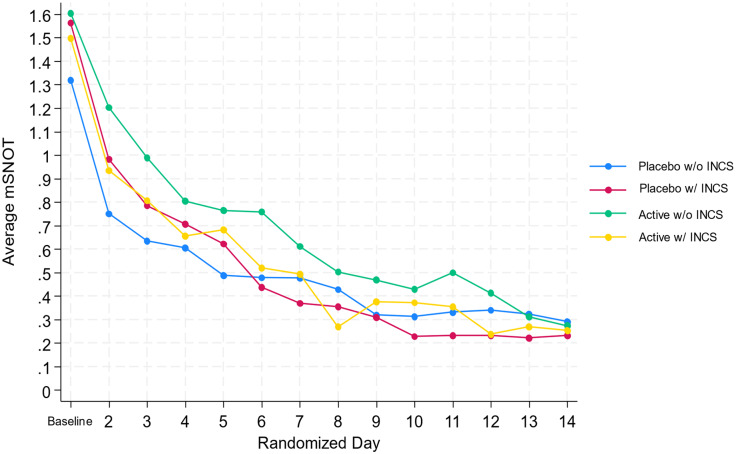
MSNOT over time.

Since the overarching goal of this pilot was not to compare differences in effectiveness between groups, we will not fully report on primary or secondary outcomes. However, it was validating to see that, as expected, all participants improved over time, and confirmed by both the mSNOT-16 and the Patient Global Impression of Change scores (see Figure 4). An important finding that these two quality-of-life scores correlated quite well, Table 4. The Patient Global Impression of Change scale takes less time to complete, and we will continue to assess correlation with the validated mSNOT-16 scores in the full-scale study.

**Table 4.** Associations between the global rating scale and mSNOT-16 scale for days E9 and R3, continuous mSNOT-16 scores by the ordered categories of the global rating scale

	Global rating categories						p-value for trend	Correlation coefficient (p-val)
**mSNOT-16**	No symptoms	A lot better	A little better	Same	A little worse	A lot worse		
Day E9 (N = 103)	0.13 (0.20) *N* = 8	0.45 (0.36) *N* = 38	1.07 (0.55) *N* = 33	1.24 (0.55) *N* = 16	1.53 (0.36) *N* = 5	1.63 (0.63) *N* = 3	<0.001	0.65 (<0.001)
Day R3 (N = 83)	0.25 (NA) *N* = 1	0.31 (0.33) *N* = 24	0.76 (0.45) *N* = 40	1.08 (0.60) *N* = 11	1.69 (0.44) *N* = 5	1.72 (0.04) *N* = 2	<0.001	0.59 (<0.001)

## Discussion

A primary goal of the feasibility phase was to evaluate recruitment and study methods prior to implementing a large clinical trial that requires significant resources. Conducting studies that are pragmatic and assess “real world” comparative effectiveness are important to helping address implementation and sustainability in busy primary care and urgent care clinics. Understanding and minimizing potential burdens on participants, clinicians, and clinic personnel are key considerations. Stakeholder input is a cornerstone of such studies.

In Results, we described changes to the study protocol that will be implemented for the larger RCT. The engagement work enabled us to better understand and reconcile issues that can often be overlooked by researchers. These include operational refinements that would not only improve participation but will help enhance the generalizability of future findings.

For example, PAB members emphasized the shift in preferences away from phone communication and willingness to increase the frequency of self-reported collection. Participant self-reports provided the opportunity to gather more information at more frequent intervals; however, each interaction was shorter than anticipated.

Review of the data collected from individuals who declined to participate, failed inclusion, or were not approached, helped the researchers reassess some inclusion and exclusion criteria. We recognized how many patients who would have otherwise qualified but were excluded from the study solely due to age (65–75 years). Extensive feedback from the SAC, DEC, and clinicians in the field challenged the requirement for participants to be sick for at least10 days to be randomized, without the consideration of “double-sickening.” For the full-scale study, procedures have been added to immediately randomize participants with double-sickening.

The other important goal for pilot is to demonstrate that one can follow the protocol and enroll and retain participants within the framework of a randomized placebo-controlled study. It is always difficult to enroll participants when their other option, routine care, means the likelihood of antibiotics being prescribed and no chance of placebo. Participants are advised to talk to their treating clinicians if they had not improved by day 3. This is consistent with IDSA guidelines that recommend switching medications if there was no improvement after day 3. While we recognize that this is indeed a challenge, we were able to enroll and deliver interventions to nearly all the participants we agreed upon within a 2-month time window. Importantly, we needed to ensure that participants would not drop out if they achieved clinical improvement before day 10. Based on focus groups and discussions with clinicians involved in the study, we based our sample size calculation for the full study on 60% being randomized. Achieving this is a significant accomplishment.

In addition to achieving our targets for recruiting, enrolling, and retaining participants, we had much higher compliance rates than anticipated. Overall compliance to the daily diary assessments was 94%, in a timeframe ranging from up to 9 days prior to and 14 days after the day of randomization. That is important as we transition to the full-scale study, as it will allow us to collect nearly all our data electronically and efficiently.

We had lower rates of saline nasal irrigation use than anticipated. To account for variations in local water quality, participants were provided with 2 gallons of distilled water, along with a standard Neti pot and saline packets. Research personnel observed that some patients were reluctant to travel from the clinic with this volume of water. Proper storage of the water after unsealing, and mixing the solution on a per-use basis also discouraged uptake. In response, changes to the protocol and actual nasal irrigation device will be implemented for the full-scale study. The newer nasal irrigation device has a built-in microfilter can be safely used with municipal tap water, avoiding the need to transport distilled water. Additionally, the PAB offered suggestions to improve the discussion and demonstration of SNI rationale and use, detailed written and video instructions, and best practices for communication training of research personnel.

We reported some pilot outcomes in this paper but as expected with the small sample, did not see any differences among the four groups. In addition to our primary outcome reported on the mSNOT-16, we also collected the global assessment of change, a 6-point scale that others have used in ARS. We found they correlated well when comparing these two scales.

We are about to undertake a transformative study in primary care. Like much of primary care, ARS is very common but understudied, with only a few small RCTs reporting heterogeneous results. This study will be nearly 10 times larger than any previous ARS study. This large sample size will allow us to look for subgroups that may benefit, understand the role of a variety of supportive care options and truly use antibiotics properly for ARS. It is clear from all the lessons we learned that we would not have been able to accomplish this without this preparatory time and the freedom to do a pilot study.

## Supporting information

10.1017/cts.2025.10175.sm001 Merenstein et al. supplementary material Merenstein et al. supplementary material

## Supplementary material

The supplementary material for this article can be found at https://doi.org/10.1017/cts.2025.10175.
