# Supplementary material for: Moving from a pilot study to large pragmatic trial in primary care settings: A study on acute rhinosinusitis
Source: J Clin Transl Sci. 2025 Nov 6;9(1):e256. doi: 10.1017/cts.2025.10175 (PMC12766510; doi:10.1017/cts.2025.10175)
Supplement: Merenstein et al. supplementary material [file S2059866125101751sup001.docx]

**APPENDIX**

**Supplemental Table 1.**

| **Inclusion and Exclusion Criteria for Pilot** |
| --- |
| Inclusion: *eligible* to participate if they are:   1. 18-65 years old; **AND** are experiencing 2. "persistent" symptoms or signs compatible with ARS or sinus infection lasting for 1-21 days without any evidence of clinical improvement (Symptoms include: facial pain or pressure, facial congestion or fullness, nasal obstruction, nasal discharge, no or reduced sense of smell, fever ≤39°C or 102°F, headache, bad smelling breath, fatigue, ear pain or pressure, and dental pain) |
| Exclusion: *ineligible* to participate for any of the following:   1. allergy or intolerance to penicillin 2. received systemic antibiotic therapy in the past 4 weeks 3. prior sinus surgery 4. complications of rhinosinusitis (facial edema, cellulitis, or orbital, meningeal or cerebral signs) 5. health care clinician determined IV (intravenous) antibiotics or hospital admission are required 6. pregnancy or breastfeeding 7. presence of a comorbidity or medication that may impair a patient’s immune response as determined by a health care clinician 8. unable to read, speak or understand English or Spanish 9. hospitalization in past 5 days 10. unable or unwilling to provide informed consent or comply with study protocol requirements 11. fever >39°C or 102°F 12. taking intranasal corticosteroids (INCS) regularly in the past two weeks |
